# Blood-Brain Barrier Permeable Chitosan Oligosaccharides Interfere with β-Amyloid Aggregation and Alleviate β-Amyloid Protein Mediated Neurotoxicity and Neuroinflammation in a Dose- and Degree of Polymerization-Dependent Manner

**DOI:** 10.3390/md18100488

**Published:** 2020-09-25

**Authors:** Limeng Zhu, Ruilian Li, Siming Jiao, Jinhua Wei, Yalu Yan, Zhuo A. Wang, Jianjun Li, Yuguang Du

**Affiliations:** 1 State Key Laboratory of Biochemical Engineering, Institute of Process Engineering, Chinese Academy of Sciences, Beijing 100190, China; lmzhu@ipe.ac.cn (L.Z.); rlli@ipe.ac.cn (R.L.); smjiao@ipe.ac.cn (S.J.); jhwei@ipe.ac.cn (J.W.); ylyan@ipe.ac.cn (Y.Y.); 2 University of Chinese Academy of Sciences, Beijing 100049, China

**Keywords:** Alzheimer’s disease, chitosan oligosaccharides, blood-brain barrier, Aβ42, aggregation, binding affinity, cytotoxicity

## Abstract

It is proven that β-amyloid (Aβ) aggregates containing cross-β-sheet structures led to oxidative stress, neuroinflammation, and neuronal loss via multiple pathways. Therefore, reduction of Aβ neurotoxicity via inhibiting aggregation of Aβ or dissociating toxic Aβ aggregates into nontoxic forms might be effective therapeutic methods for Alzheimer’s disease (AD) treatment. This study was designed to explore interference of chitosan oligosaccharides (COS) on β-(1-42)-amyloid protein (Aβ42) aggregation and Aβ42-induced cytotoxicity. Here it was demonstrated that COS showed good blood-brain barrier (BBB) penetration ability in vitro and in vivo. The experimental results showed that COS efficiently interfered with Aβ42 aggregation in dose- and degree of polymerization (DP)-dependent manners, and COS monomer with DP6 showed the best effect on preventing conformational transition into β-sheet-rich structures. Based on the binding affinity analysis by microscale thermophoresis (MST), it was confirmed that COS could directly bind with Aβ42 in a DP-dependent manner. Our findings demonstrated that different performance of COS monomers with different DPs against Aβ42 assembly was, to some extent, attributable to their different binding capacities with Aβ42. As a result, COS significantly ameliorated Aβ42-induced cytotoxicity. Taken together, our studies would point towards a potential role of COS in treatment of AD.

## 1. Introduction

Alzheimer’s disease (AD) is a chronic neurodegenerative disease characterized by co-existence of extracellular senile plaques (SP) of accumulated insoluble Aβ and intracellular neurofibrillary tangles (NFT) of hyperphosphorylated tau protein [1,2]. Formation of SP is a multiple and complex self-assembly process of Aβ peptides [3]. The amyloid cascade hypothesis presumes that Aβ self-assembles into well-ordered aggregates containing β-sheet-rich structures, leading to dysfunction and neurodegeneration of the central nervous system (CNS) [4,5,6]. Chemicals, which could intervene with Aβ assembly and lead to a reduction of the characteristic cross-β-sheet structures, can inhibit Aβ induced neurotoxicity in vitro and reverse cognitive impairment in vivo [7,8,9]. Therefore, reduction of Aβ neurotoxicity via inhibiting aggregation of Aβ might be one of the promising therapeutic methods for AD treatment.

Until now, numerous inhibitors, including antibodies, peptides, and synthetic and natural compounds, have been tested, but only a few of them are suitable for clinical trials due to some limitations [10,11]. For example, peptide-based inhibitors can be easily degraded by enzymes and show poor BBB permeability [12]. Moreover, many inhibitor candidates for the treatment of AD failed in different phases of clinical trials. Scyllo-inositol, a potent Aβ aggregation inhibitor [13], failed in a phase 2 randomized trial involving 353 Alzheimer patients due to the non-significantly clinical efficacy outcomes [14]. At present, more attention has been paid to small chemical compounds that are derived from natural products because of their high permeability through the BBB, ease of accessibility, and low cytotoxicity [15,16]. Chitosan oligosaccharides (COS), linked by β-(1-4) bonds through d-glucosamine and *N*-acetyl-d-glucosamine, are mixtures of oligomers with a degree of polymerization (DP) of <20 and an average molecular weight (MW) of <3.9 kDa, which are produced enzymatically or chemically from chitosan [17]. Due to their higher water solubility, low cytotoxicity, and easy degradability, COS have gained considerable attention at present [18]. Recently, researchers have confirmed that COS possess various physiological activities, such as anti-oxidation, anti-inflammation, immune regulation, and neuroprotective properties [19]. The neuroprotective activities of COS include inhibition of β-secretase and acetylcholinesterase, anti-neuroinflammation, anti-excitotoxic effects, and anti-apoptosis [20]. However, these studies were mainly verified by in vitro cellular models, and whether COS could penetrate through the BBB and reach the brain to exert their neuroprotective effects directly remains elusive. Unlike most oligosaccharides, COS are the only natural ones with positive charges, which allow them to bind easily with other molecules via hydrogen bonds and electrostatic interactions, this property is responsible for many of their observed biological activities [21]. Meanwhile, it remains unclear whether COS could directly bind with Aβ to prevent conformational transition and self-assembly of Aβ, thereby alleviate amyloid-induced cytotoxicity considering their neuroprotective properties.

In this study, whether COS could penetrate through the BBB in vitro and in vivo was examined. The interference effects of COS on conformational transition and morphological changes of Aβ42 were conducted by circular dichroism (CD) spectroscopy and transmission electron microscopy (TEM), respectively, to explore the direct effects of COS on Aβ42 assembly. Considering that COS monomers with different DPs may have different biological activities, the inhibition effects of COS monomers with different DPs on Aβ42 fibrillation were also evaluated. Microscale thermophoresis (MST) was used to detect whether COS and Aβ42 could directly bind with each other and the binding affinities between COS monomers with different DPs and Aβ42 were further determined to explore the potential mechanism involved. Meanwhile, Aβ42-mediated neurotoxicity and neuroinflammation were also investigated.

## 2. Results

### 2.1. Structural Characterization of COS

**Figure 1:**
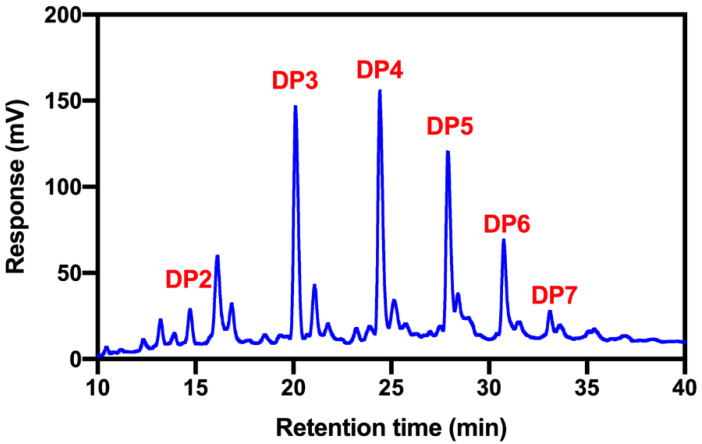
HPLC analysis of COS mixture with a charged aerosol detector. COS monomers with DPs in the range of 2–7. Their respective weight percentages were determined as follows: 3.69% ± 0.23% (DP2), 29.23% ± 0.40% (DP3), 30.81% ± 0.62% (DP4), 22.25% ± 0.35% (DP5), 11.64% ± 0.28% (DP6), and 2.37 ± 0.25% (DP7). Data were expressed as mean ± SEM.

The deacetylation degree of COS was determined to be 93% by ^1^H NMR spectroscopy based on a method described previously [22] (Supplementary Figure S1). The DPs of COS were determined to be in the range of 2–7, and the weight percentages of COS monomers with different DPs were quantified by HPLC analysis with a charged aerosol detector to be 3.69% ± 0.23% (DP = 2), 29.23% ± 0.40% (DP = 3), 30.81% ± 0.62% (DP = 4), 22.25% ± 0.35% (DP = 5), 11.64% ± 0.28% (DP = 6) and 2.37 ± 0.25% (DP = 7), respectively (Figure 1).

### 2.2. Permeation of COS across the Blood Brain Barrier In Vitro and In Vivo

**Figure 2:**
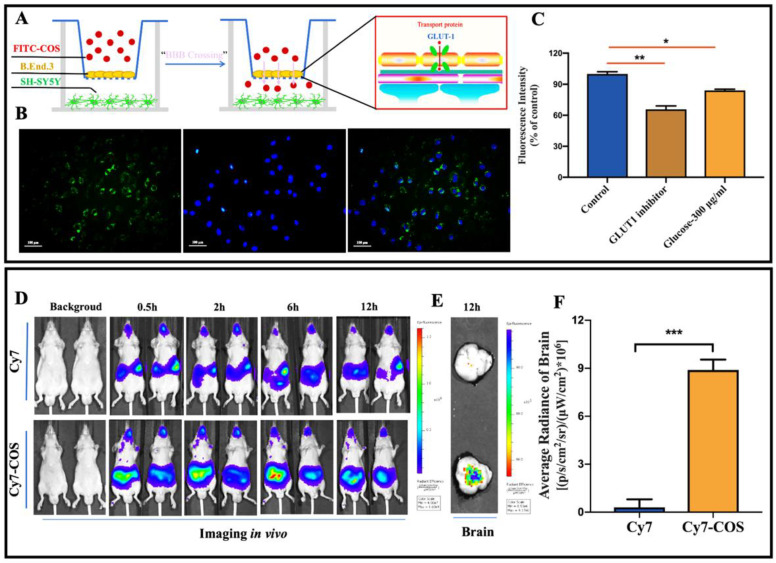
Characterization of the BBB penetration ability of COS both in vitro and in vivo. (**A**) The schematic illustration of in vitro BBB Transwell model. (**B**) Trans-well model for evaluating BBB penetration of COS in vitro. bEnd.3 cells were incubated with FITC-labeled COS in the upper insert for 6 h at 37 °C, and the fluorescence intensity was detected by fluorescence microscope (scale bar, 200 μm. Green for FITC, excitation wavelength: 490 nm; emission wavelength: 525 nm. Blue for nucleus, excitation wavelength: 359 nm; emission wavelength 461 nm. Data were expressed as mean ± SEM of three separate experiments). (**C**) Suppressive effects of WZB117 and glucose on BBB permeability of COS. The mean fluorescence intensity was quantitatively analyzed. (**D**) The biodistribution of Cy7 labeled COS in mice. Cy7-labeled COS and Cy7 NHS were administrated to nude mice via gavage. The fluorescence was detected by IVIS spectrum in vivo imaging system at different time points. (**E**) IVIS spectrum of brains of sacrificed mice. Mice were sacrificed at 12 h post administration and the fluorescence in brains was detected. (**F**) Average radiance of brains detected by IVIS spectrum in vivo imaging system. Data were expressed as the mean ± SEM of three separate mice; * *p* < 0.05, ** *p* < 0.01, *** *p* < 0.001.

To determine permeation of COS across the BBB, a well-established trans well in vitro platform was used to model the attributes and functions of BBB (Figure 2A). The barrier function of the bEnd.3 layer was assessed by measuring the *P_app_* values of sodium fluorescein and fluorescein isothiocyanate (FITC)-labeled Dextran with different molecular weights (10 kDa, and 40 kDa) across the bEnd.3 cell monolayer. The *P_app_* value of FITC-dextran (10 kDa) was reduced gradually with extension of incubation time, and was stable at about 3.7 × 10^−6^ cm s^−1^ after 6 days of cultivation (Supplementary Figure S2A), proving that the endothelial layer became fully dense on the sixth day. In addition, the *P_app_* value of FITC-dextran (40 kDa) reached 2.9 × 10^−7^ cm s^−1^ on the sixth day, lower than that of sodium fluorescein and FITC-dextran (10 kDa), suggesting that *P_app_* was correlated with molecular weights (Supplementary, Figure 2B). Moreover, one of the tight junctional proteins, occludin, expressed on bEnd.3 cells was examined by immunofluorescent staining. As shown in Supplementary Figure S2C, tight junctions in these cells appeared as near-continuous rings localized to the periphery of each cell. These data demonstrated that the bEnd.3 cells on the top chamber membrane formed a size-selective barrier for trans-endothelial transport after 6 days of incubation.

To investigate the BBB penetration ability of COS, the fluorescence intensity in the lower chamber was detected. As shown in Figure 2B, FITC-labeled COS penetrated through monolayer of bEnd.3 cells and were distributed around or inside SH-SY5Y cells, suggesting that COS could easily penetrate across the BBB in vitro model. To determine whether any transporters are required for their BBB penetration, inhibitor of GLUT1 (WZB117) was added. Compared to the control group, reduced fluorescence intensity of FITC-COS was observed in the lower chamber (*p* < 0.01), indicating that WZB117 decreased the permeation ability of COS across the BBB (Figure 2C). Meanwhile, considering that GLUT1 was one of the influx glucose transporters, competitive inhibition of glucose with COS was examined, and the results showed that the fluorescence intensity was significantly lower due to glucose administration (*p* < 0.05) (Figure 2C). These results demonstrated that GLUT1 was one of the transporters accounting for BBB penetration of COS.

To study biodistribution of COS and verify the permeation across the BBB in vivo, cyanine 7 N-hydroxy succinimide ester (Cy7-NHS) and Cy7-labeled COS were administrated by gavage and the fluorescence intensity was measured using an IVIS spectrum in vivo imaging system (Figure 2D). Consistently, Cy7-labeled COS were observed in the brains of mice sacrificed at 12 h post administration, confirming that COS showed good BBB penetration ability in vivo (Figure 2E,F).

### 2.3. Inhibition Effect on Aβ42 Fibrillation and Disaggregation Effect on Preformed Aβ42 Fibrils

**Figure 3:**
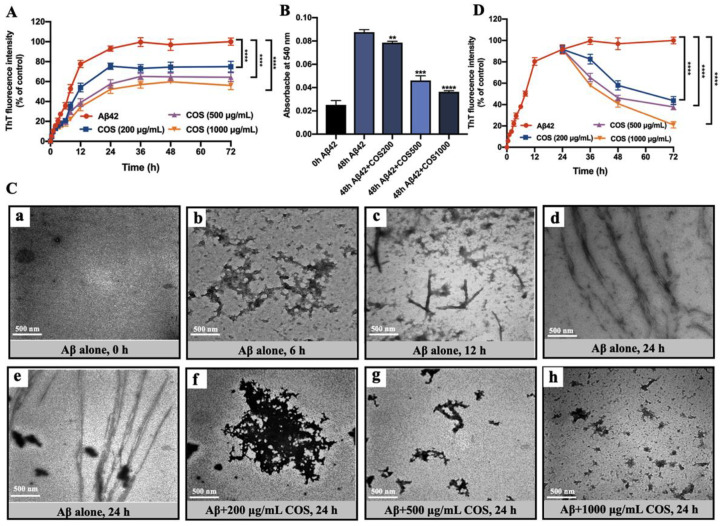
Effects of COS on Aβ42 assembly. (**A**) Inhibition assay for Aβ42 fibrils intervene with COS. Formation of Aβ42 (20 μM) fibrils was monitored by time-dependent ThT fluorescence with or without different concentrations of COS (200, 500, and 1000 μg/mL, respectively). Fluorescence intensity was measured at excitation and emission wavelengths of 440 and 480 nm, respectively. (**B**) CR spectral shift assay to quantify Aβ42 aggregates. Absorbance changes of CR spectra for 20 μM Aβ42 incubated with or without different concentrations of COS (200, 500, and 1000 μg/mL, respectively) for 48 h, were compared with Aβ42 alone. (**C**) The morphological changes of Aβ42 aggregates in the presence or absence of COS. The effects of COS on the morphologies of Aβ42 aggregates were visualized by a Hitachi H7500 TEM at 80 kV. Representative TEM images of 20 μM Aβ42 monomer soon after prepared (**a**) and oligomers or fibrils formed by monomeric Aβ42 after 6 h (**b**), 12 h (**c**), and 24 h (**d**) incubation at 37 °C without agitation respectively; (**e**) TEM images of monomeric 20 μM Aβ42 co-incubated with 200 μg/mL (**f**), 500 μg/mL (**g**), and 1000 μg/mL (**h**) COS for 24 h at 37 °C were also visualized. The scale bar (500 nm) is shown on the lower left of the images. (**D**) Effects of COS on dissociating toxic Aβ42 to form off-pathway aggregates. 20 μM Aβ42 was pre-incubated at 37 °C for 24 h. Preformed Aβ42 fibrils were monitored by ThT fluorescence in the absence and presence of COS at different concentrations (200, 500, and 1000 μg/mL, respectively) at different time points with excitation and emission wavelengths of 440 and 480 nm. Data are represented as mean ± SEM of three separate experiments; ** *p* < 0.01, *** *p* < 0.001, **** *p* < 0.0001, vs. the Aβ42 alone group.

The inhibition effects of COS on Aβ42 fibrillation were examined by ThT fluorescence assay and TEM. As shown in Figure 3A, ThT fluorescence showed significant decrease in the plateau phase by ~25% with administration of 200 μg/mL COS compared to the Aβ42 group (*p* < 0.0001), suggesting a lower growth rate and a lower content of β-sheet structures in the presence of COS. When the concentrations of COS were further increased to 500 and 1000 μg/mL, stronger inhibition was observed, with the final fluorescence intensity being reduced by ~36% and ~44%, respectively (*p* < 0.0001) (Figure 3A). These results indicated that COS exerted obvious inhibiting effects on Aβ42 aggregation in a dose-dependent manner (Figure 3A). Consistent with the ThT fluorescence results, CR absorbance at 540 nm in sample of Aβ42 alone was significantly increased after incubated for 48 h, whereas intervention with COS obviously decreased CR absorbance in a dose-dependent manner (*p* < 0.01, 0.001, or 0.0001) (Figure 3B). Meanwhile, TEM was used to further investigate the effects of COS on the morphology of Aβ42 aggregates. As shown in the image a of Figure 3C, the TEM image of monomeric Aβ42 soon after prepared confirmed the absence of aggregated Aβ42 in these preparations. After 6 h of incubation, small and globular aggregates were observed in Aβ42 solution (Image b of Figure 3C). After 12 h, short, needle-like and irregular protofibrils were produced (Image c of Figure 3C), and after 24 h, these irregular aggregates appeared to have agglomerated into numerous mature and bundled Aβ42 fibrils (Image d of Figure 3C). However, in the presence of COS, the morphology of Aβ42 aggregates was dramatically changed compared to Aβ42 alone. After 24 h of co-incubation with COS, shorter and thinner fibrils or completely irregular and amorphous aggregates was formed rather that bundled fibrils (Images f–h of Figure 3C). These observations suggested that Aβ42 oligomers were prevented from further growing into mature fibrils by COS in a dose-dependent manner.

To investigate whether COS could disaggregate preformed mature Aβ42 fibrils, 20 μM Aβ42 was pre-incubated at 37 °C for 24 h to form mature fibrils. As shown in Figure 3D, the ThT fluorescence intensity curve showed a plateau phase after 24 h, suggesting that the fibrillation process reached an equilibrium and the pre-incubation time was long enough to form mature fibrils. However, a remarkable reduction in ThT fluorescence intensity of Aβ42 fibrils was observed with the addition of COS in a dose-dependent manner as compared with Aβ42 alone (*p* < 0.0001). Our results indicated that COS could promote disaggregation of preformed mature Aβ42 fibrils in a dose-dependent manner (Figure 3D).

### 2.4. Changes of Secondary Structures of Aβ42 Affected by COS Mixture and COS Monomers with Different DPs

**Figure 4:**
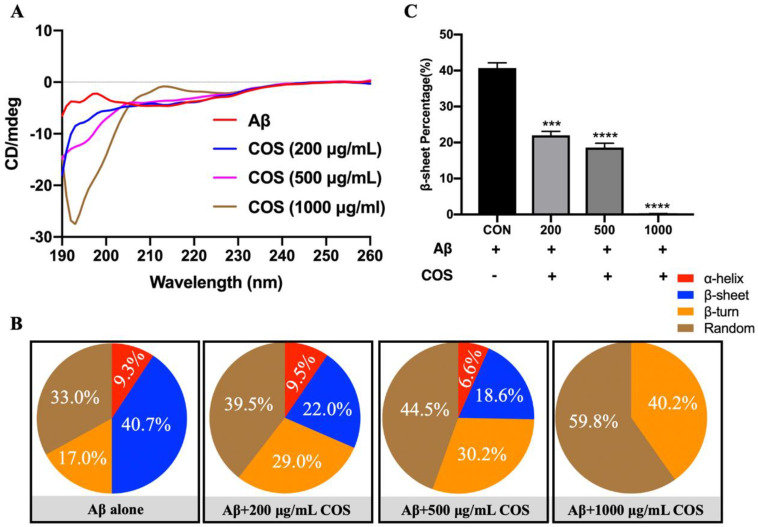
Effects of COS mixture on formation of Aβ42 fibrils measured by CD spectra. (**A**) CD spectra for different treatment groups. The CD spectra of 20 μM Aβ42 incubated with or without different concentrations of COS (200, 500, and 1000 μg/mL, respectively) were recorded at 24 h in the spectral range of 190–260 nm. Data were the average of three runs and represented as colored curves after being smoothed. (**B**) Proportions of different secondary structures of Aβ42 fibrils analyzed by the Protein Secondary Structure Estimation Program. (**C**) Changes of β-sheet structures after administration of COS. COS blocked formation of β-sheet structures during Aβ42 aggregation. Data are represented as mean ± SEM. *** *p* < 0.001, **** *p* < 0.0001 vs. Aβ42 alone.

CD spectroscopy was used to investigate the effects of COS on conformational transition of Aβ42 (Figure 4). After incubated for 24 h, the CD spectrum of Aβ42 showed a positive peak at around 195 nm and a negative valley at around 214 nm (Figure 4A), which corresponded to a mixture of α-helix and β-sheet structures. From Figure 4B, the secondary structures of pure Aβ42 fibrils were mainly composed of α-helix (about 9.3%), β-sheet (about 40.7%), β-turn (about 17.0%), and random coil structures (about 33.0%). However, the positive peak appeared at around 195 nm diminished gradually which indicated that the percentage of β-sheet structures were obviously decreased with the addition of COS as concentration was increased (*p* < 0.001 or 0.0001) (Figure 4C). By contrast, 22.0% of β-sheet structures were formed in the presence of 200 μg/mL COS, which was less than those of the Aβ42 group (*p* < 0.001). When the concentration of COS was increased to 1000 μg/mL, the β-sheet structures almost completely disappeared after 24 h of incubation (Figure 4B). The decreased β-sheet structures with a concomitant increase in random coil structures indicated the remarkable inhibitory effects of COS on formation of amyloid fibrils (Figure 4B).

**Figure 5:**
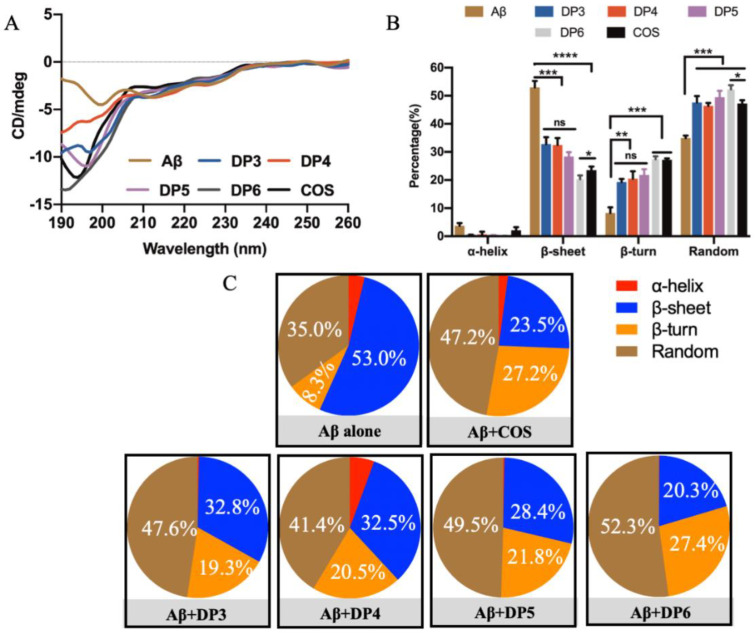
Effects of DPs of COS monomers on formation of Aβ42 fibrils measured by CD spectrum. (**A**) CD spectra obtained in different treatment groups. The CD spectra of 20 μM Aβ42 incubated with or without 200 μg/mL COS with different DPs (3–6) were recorded after 24 h incubation in the spectral range of 190–260 nm. Data were the average of three runs and represented as colored curves after being smoothed. (**B**) Changes of β-sheet structures after administration of COS monomers with different DPs. COS blocked formation of β-sheet structures during Aβ42 aggregation in a DP-dependent manner. And COS monomer with DP6 was the most effective component in inhibiting formation of Aβ42 β-sheet structures. (**C**) Proportions of different secondary structures of Aβ42 fibrils analyzed by the Protein Secondary Structure Estimation Program. * *p* < 0.05, ** *p* < 0.01, *** *p* < 0.001, **** *p* < 0.0001.

In addition, effects of COS monomers with different DPs on conformational transitions of Aβ42 were also investigated (Figure 5A). The concentrations of COS monomers used in CD spectroscopy assay was consistent with their respective weight percentages determined by HPLC. The result showed that the typical CD spectra of Aβ42 were changed extremely by treatment with COS monomers with different DPs (Figure 5A). And the percentage of β-sheet in Aβ42 was significantly reduced in the presence of COS in a DP-dependent manner (*p* < 0.001 or 0.0001) (Figure 5B,C). It is remarkably notable that COS monomer with DP6 was the most effective one in inhibiting formation of β-sheet structures (Figure 5B). Therefore, it was concluded that COS could reduce the β-sheet contents in a dose- and DP-dependent manner.

### 2.5. Interactions of Aβ42 with COS Mixture or COS Monomers with Different DPs by MST

**Figure 6:**
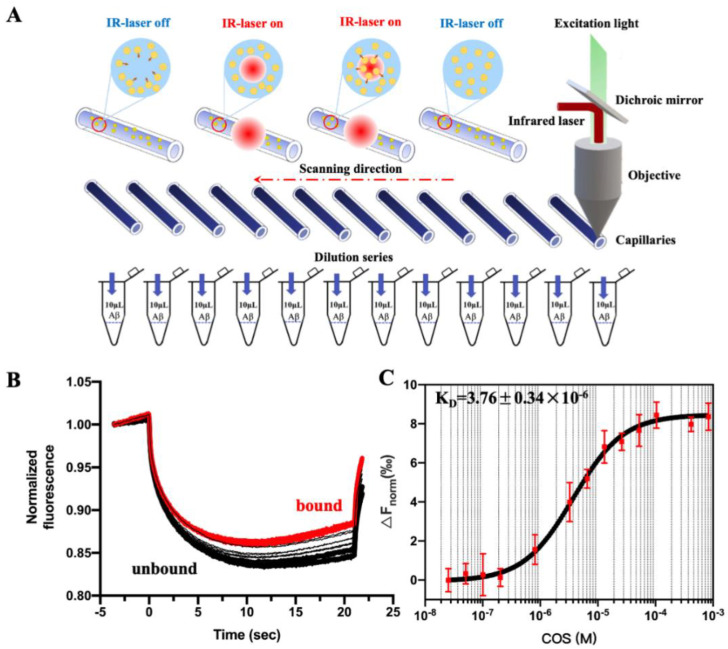
Monitoring binding events between COS and Aβ42. (**A**) The physical principle and solutions preparation for MST test. Potential interaction partners are incubated in a glass capillary. At a specific temperature, an infrared laser generates a temperature gradient. Interaction partners which have formed a complex move more slowly in this gradient than free interaction ones. A binding curve can be calculated from the difference between the fluorescence signals of both possible states, from which a binding constant can be derived [23]. (**B**) Relative fluorescence intensity between the bound and unbound state. The thermophoretic movement of a fluorescently-labeled molecule (black trace; ‘‘unbound’’) changes upon binding to a non-fluorescent ligand (red trace; ‘‘bound’’), resulting in different traces. (**C**) A typical MST curve for interaction of COS with Aβ42. A *K_D_* of 3.76 ± 0.34 μM was determined for this interaction employing standard data analysis with MO. Affinity Analysis Software. The graphs displayed data from three independent measurements. Error bars represented the SEM.

To determine the interaction of COS with Aβ42, microscale thermophoresis was carried out (Figure 6A). Pretests were performed to investigate adsorption of cyanine 5 (Cy5)-labeled Aβ42 onto standard-MST capillary walls prior to the MST experiments. The results showed that Cy5 labeled Aβ42 did not self-aggregate or absorb onto capillary walls in MST buffer (data not show). For the subsequent experiments, Cy5-labeled Aβ42 was used at a concentration of 4 μM, while non-fluorescent labeled COS mixture as binding ligands were titrated with concentrations between 800 μM and 12.2 nM. Relative fluorescence intensity between the bound and unbound state between COS mixture and Aβ42 was recorded (Figure 6B), and a typical MST curve showed a single binding event with *K_D_* of 3.76 ± 0.34 μM for the binding of Aβ42 with the COS mixture (Figure 6C).

**Figure 7:**
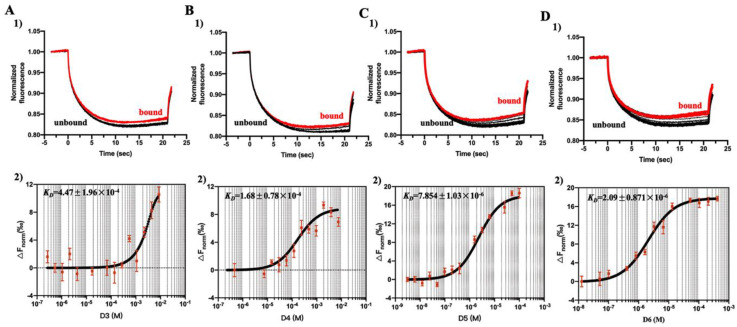
MST analysis of interaction of Aβ42 with COS monomers with different DPs. Relative fluorescence intensity (**1**) and MST curve (**2**) for Aβ42 binding with DP3 (**A**), DP4 (**B**), DP5 (**C**), and DP6 (**D**). The changes in MST signals were fitted (blank line) to yield *K_D_* values of (**A**) 447 ± 196 μM (DP3), (**B**) 168 ± 78 μM (DP4), (**C**) 7.854 ± 1.03 μM (DP5), and (**D**) 2.09 ± 0.87 μM (DP6), respectively. Error bars indicate the SEM; *n* = 3.

Apart from sequence homology, oligosaccharide-peptide interactions are also affected by conformation and DPs of oligosaccharides. For this reason, the same procedures were repeated for COS monomers with DPs in the range of 3 to 6. The MST traces and the binding curves were shown in Figure 7, and the binding affinities (*K_D_*) and constants (*K_a_*= 1*/K_D_*) of the different ligands with Aβ42 were shown in Supplementary Table S1. The binding affinities showed an obvious DP-dependent manner. Among all the ligands, COS monomer with DP6 showed the strongest binding affinity to Aβ42, which was indicated by its lowest *K_D_* (2.09 ± 0.87 μM) and highest *K_a_* (5.95 ± 2.48 × 10^5^ M^−1^) (Supplementary Table S1). Meanwhile, the results of binding affinity between Aβ42 and COS monomers with different DPs obtained from MST were consistent with those of the CD spectra.

### 2.6. Alleviation of Amyloid-Induced Cytotoxicity by COS

**Figure 8:**
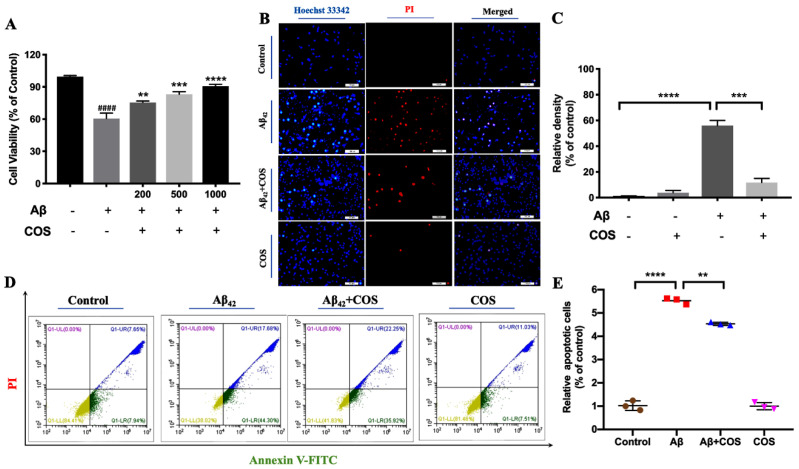
Alleviation of amyloid-induced cytotoxicity by COS. (**A**) Cell viability detected by MTT assay. Cells were treated with 5 μM Aβ42 with or without different concentrations of COS for 24 h, and the cell viability was detected by MTT assay. (**B**) Hoechst 33342 and PI fluorescence photomicrographs of groups intervened with different concentrations of COS. In contrast with the control group, the cell nuclei of Aβ42 group were smaller and more brightly stained because of the presence of condensed chromatin. COS administration markedly alleviated Aβ42-induced cell apoptosis. (**C**) Quantitative analysis of the mean fluorescence intensity of bright blue area. (**D**) Cell apoptosis determined with Annexin-V/Pl staining by flow cytometry analysis. (**E**) Quantitative analysis of the apoptotic cell in the lower right (LR) quadrant. Data were represented as mean ± SEM of three separate experiments; ** *p* < 0.01, *** *p* < 0.001, and **** *p* < 0.0001 vs. Aβ42 group; #### *p* < 0.0001 vs. control group.

To determine cytotoxicity of Aβ42 intervened by COS to human neuroblastoma SH-SY5Y cells, MTT, Hoechst 33342 immunofluorescent staining and Annexin V/PI assays were conducted to measure cell viability and apoptosis. When SH-SY5Y cells were treated with different concentrations of COS, no significant changes were observed in cell viability, suggesting that COS were not toxic to SH-SY5Y cells (Supplementary Figure S3A). As shown in Figure 8A, Aβ42, which was allowed to oligomerize and fibrillize within 24 h in cell culture media, remarkable decrease in cell viability from 100% to 60.1% was observed compared with the untreated control group (*p* < 0.001), whereas COS significantly attenuated Aβ42-induced cytotoxicity in a dose-dependent manner (*p* < 0.01, 0.001, or 0.000). For example, cell viability reached the highest value of about 90.5% after co-incubated with 1000 μg/mL COS for 24 h (*p* < 0.0001). Therefore, the results indicated that Aβ42 exhibited low cytotoxicity after intervening with COS.

Moreover, double immunofluorescent staining by Hoechst 33342 and PI was also used to determine the effects of COS on Aβ42-induced cell apoptosis. Compared to the control group, nuclei of the 10 μM Aβ42 treated group were smaller and more brightly stained because of the presence of condensed chromatin (Figure 8B), suggesting that Aβ42 treatment led to cell apoptosis (*p* < 0.0001). Consistently, when Aβ42 mixed with COS was added to cell culture, COS markedly alleviated Aβ42-induced cell apoptosis (*p* < 0.001) (Figure 8B,C). Annexin V/PI staining was also detected by flow cytometry to monitor apoptotic status of SH-SY5Y cells. As shown in Figure 8D, the addition of COS protected cells from toxicity induced by Aβ42 (*p* < 0.001) (Figure 8D,E).

### 2.7. Attenuation of Oxidative Stress and Reduction of Release of Inflammatory Cytokines in BV2 Cells Intervened with COS

**Figure 9:**
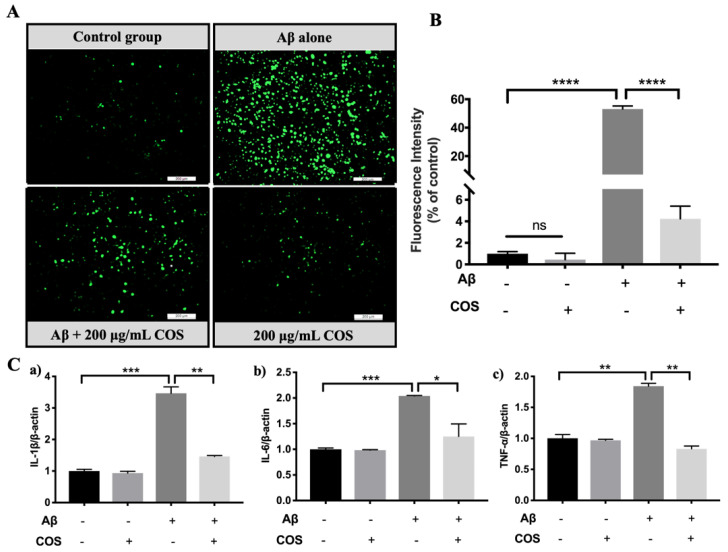
Inhibition effects of COS on Aβ42-induced activation of microglia, release of inflammatory cytokines, and production of ROS. (**A**) ROS production detected by DCFH-DA in BV-2 cells. The intracellular levels of fluorescence were determined using a fluorescence microscopy. (**B**) Quantitative analysis of the mean fluorescence intensity in BV2 cells. (**C**) The expression levels of inflammatory cytokines IL-1β (**a**), IL-6 (**b**), and TNF-α (**c**) measured by RT-PCR analysis. Data were represented as mean ± SEM of three separate experiments; * *p* < 0.05, ** *p* < 0.01, *** *p* < 0.001, **** *p* < 0.0001.

To investigate the effects of COS on Aβ42-induced oxidative stress and neuroinflammation, microglial BV2 cells were stimulated with Aβ42 in the presence or absence of COS, and reactive oxygen species (ROS) and pro-inflammatory cytokines were analyzed. The effects of COS on ROS production were illustrated by 2,7-Dichlorodi-hydrofluorescein diacetate (DCFH-DA) fluorescent probe. As shown in Figure 9A, the green fluorescence was markedly stronger in the Aβ42 treated group than the untreated control group, indicating an increased ROS level induced by Aβ42 (*p* < 0.0001). However, the addition of 200 μg/mL COS significantly suppressed generation of Aβ42-induced ROS which was illustrated by the weakened green fluorescence intensity (*p* < 0.0001) (Figure 9B). Our results indicated that COS significantly inhibited the generation of ROS induced by Aβ42 oligomers.

To further determine the effects of COS on Aβ42-induced neuroinflammation, the mRNA levels of pro-inflammatory cytokines in BV2 cell were analyzed. Consistently, incubation with Aβ42 oligomers remarkably increased the transcription levels of IL-1β, TNF-α and IL-6 in BV2 cells (Figure 9C), whereas co-incubation of Aβ42 oligomers with 200 μg/mL COS significantly attenuated the Aβ42-induced increase in those of proinflammatory cytokines (*p* < 0.05, 0.01, or 0.001). Collectively, these results demonstrated that COS intervention significantly suppressed production of proinflammatory cytokines and oxidative stress induced by Aβ42.

## 3. Discussion

**Figure 10:**
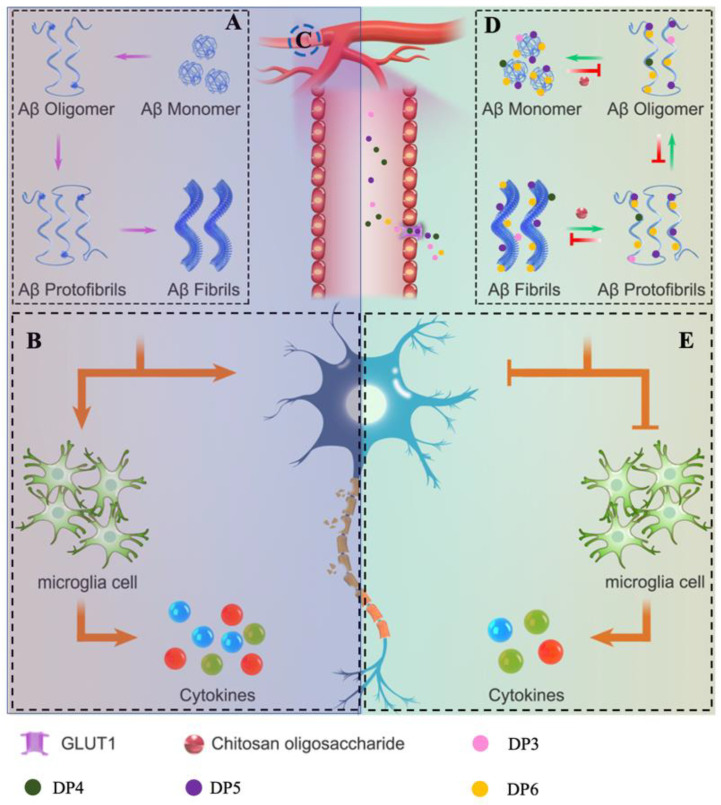
Schematic diagram showing how COS interfered with Aβ42 aggregation and alleviated Aβ42-induced cytotoxicity. (**A**) Schematic representation of the process of Aβ aggregation. (**B**) Aβ self-assembles into well-ordered aggregates containing β-sheet-rich structures, leading to neuroinflammation and neuronal damage. (**C**) COS could penetrate through BBB and GLUT1 was one of the transporters accounting for their penetration. (**D**) COS could bind with Aβ42 peptide directly and inhibit Aβ42 assembly in a dose- and DP-dependent manner. (**E**) COS significantly alleviated amyloid-induced neuronal damage and release of inflammatory cytokines via interfering with Aβ42 aggregation.

AD is a chronic neurodegenerative disease of which the exact pathogenesis remains unknown [24,25]. Recent studies have suggested that conformational changes of Aβ aggregates into structures with high β-sheet contents, including oligomers and fibrils in particular, are the main neurotoxins in AD pathogenesis [26,27]. It is proven that Aβ aggregates containing cross-β-sheet structures lead to oxidative stress, neuroinflammation, and neuronal loss via multiple pathways [3,28]. Therefore, reduction of Aβ neurotoxicity via inhibiting aggregation of Aβ or dissociating toxic Aβ aggregates into nontoxic forms might be effective therapeutic methods for AD prevention. In this study, we demonstrated that COS could easily penetrate through BBB in vitro and in vivo and bind with Aβ42 directly, which further interfering conformational changes of Aβ42 aggregates. As a consequence, COS showed efficient inhibition effects on Aβ42 fibrillation and disaggregation effect on preformed Aβ42 fibrils in dose- and DP-dependent manners. Meanwhile, COS significantly alleviated amyloid-induced apoptosis, oxidative stress and release of inflammatory cytokines. Collectively, these findings indicated that COS has potential applications for prevention or treatment of AD (Figure 10).

To inhibit Aβ aggregation and attenuate Aβ induced cytotoxicity, numerous inhibitors including antibodies, peptides, and synthetic or natural compounds have been tested, but only a few agents are suitable for clinical trials [11]. For clinical applications, inhibitors of Aβ aggregation must resist enzymatic degradation and easily cross the BBB, and not induce inflammation, toxicity, and other adverse immune responses [29].

COS, oligosaccharides of chitosan, have received considerable attention as functional, renewable, nontoxic, and biodegradable natural ones for diverse applications, especially in pharmaceutics [21]. Recently, multiple lines of evidences have suggested that COS possess good neuroprotective properties, such as inhibitory activities towards β-secretase and acetylcholinesterase, anti-neuroinflammation, and anti-apoptosis [20,30,31]. However, these studies were mainly verified by in vitro cellular models, and whether COS can reach the brain to exert their neuroprotective effects directly remains elusive. BBB is an impediment for the delivery of therapeutic agents to the brain [32]. In this study, to determine the permeation ability of COS across the BBB in vitro, a single layer of brain microvascular bEnd.3 cells as a BBB model in vitro was used (Figure 2A). The results indicated that COS showed good BBB penetration ability. The conclusion was further confirmed by the results of biodistribution of COS in mice through detecting the fluorescence intensity of Cy7-labeled COS using an in vivo imaging system (Figure 2E,F). According to the previous studies, the most efficient approach for delivery of CNS drugs is via the selective endogenous transport mechanisms like GLUT1, L-type amino acid transporter 1 (LAT-1), and transferrin receptor (TfR) [33,34]. GLUT-1 is expressed selectively at a high level in the BBB and is the energy-independent, facilitative transporter of glucose into the brain [35]. Growing evidence has suggested that GLUT-1 mediates the transport of some CNS drugs across the BBB as a delivery system, such as glycosylated neuropeptides, low molecular weight heparin, and d-glucose derivatives [36]. In our study, it was observed that the GLUT1 inhibitor WZB-117 and competitive inhibition of glucose on GLUT1 mediated transportation significantly decreased the uptakes of the BBB towards COS in vitro (Figure 2D). These results indicated that GLUT1 was one of the transporters accounting for COS penetration of the BBB.

Based on our obtained results, it is clear that COS had marked inhibitory effects on Aβ42 fibrillation and disaggregation effect on preformed Aβ42 fibrils by interfering conformational changes of Aβ42 aggregates in a dose-dependent manner (Figure 3 and Figure 4). In general, it is important to mention that COS monomers with different DPs might show different biological activities according to the previous studies [17]. However, the effects of COS monomers with different DPs on Aβ42 aggregation was unknown. Herein, the inhibitory effects of COS monomers with different DPs on formation of Aβ42 fibrils were studied. Interestingly, the obtained results from CD analyses demonstrated that β-sheet structures of Aβ42 was attenuated significantly by COS monomers in a DP-dependent manner. Additionally, COS monomer with DP6 was the most effective one in inhibiting the formation of β-sheet structures (Figure 5).

However, how could COS act on Aβ42 peptide directly and interfere with Aβ42 aggregation in a DP-dependent manner? Unlike most oligosaccharides, COS are the only natural ones with positive charges, which allows them to bind easily with other molecules, and this property is responsible for many of the observed biological activities [21]. Therefore, understanding the details of how COS interacts with Aβ42 is of great importance for exploring the involved mechanism. Several biophysical approaches, including isothermal titration calorimetry, dynamic light scattering, and surface plasmon resonance, do allow to investigate biomolecular interactions [23]. In this study, MST was used for quantitative analysis of interactions between Aβ42 and COS. The results of the MST studies concluded that the COS mixture could bind with Aβ42 directly (Figure 6). Apart from sequence homology, oligosaccharide-peptide interactions are also affected by conformation and DPs of oligosaccharides, which prompted us to determine if the DP-dependent manner was due to the different binding affinities between COS monomers with different DPs and Aβ42. Previous studies showed that COS monomers with different DPs possessed different binding capacities against lymphocyte surface receptor, namely complement III receptor (CR3) [37]. Herein, the binding affinities of Aβ42 towards COS monomers with different DPs were measured by MST. The binding affinities between them showed a DP-dependent manner. Among all ligands, COS monomer with DP6 showed the strongest binding capacity with Aβ42 (Figure 6; Supplementary Table S1). These results were consistent with those of CD analyses. Taken together, our findings collectively highlighted the possible mechanisms of COS in inhibition of Aβ42 fibrillation and disaggregation of preformed Aβ42 fibers were that COS could bind with Aβ42 directly and subsequently interfere with Aβ42 aggregation in a dose- and DP-dependent manner. Moreover, the MST results gave us a good explanation that the DP-dependent manner was, to some extent, attributable to their different binding affinities towards Aβ42.

However, why COS monomers exhibited different binding affinities towards Aβ42 remains unclear. COS mainly have two types of reactive functional groups, amino groups as well as both primary and secondary hydroxyl groups at the C-2, C-3, and C-6 positions. The amino and hydroxyl groups make them easily form intra- and inter-molecular hydrogen bonds [18]. The numbers of exposed amino and hydroxyl groups of COS monomers with higher DPs are more than those of COS monomers with lower DPs. Therefore, one potential explanation for different binding affinities of COS monomers with Aβ42 might due to the different amount of active binding sites. Meanwhile, COS monomers with higher DPs might have more active binding sites, and then interfere with Aβ42 aggregation more efficiently due to their higher binding affinity. The molecular weight of DP6 is higher than that of other DPs and, relatively, a greater amount of active binding sites was available. Therefore, DP6 has more active binding sites to interact with Aβ42. Consequently, DP6 exhibits a higher binding affinity to Aβ42, and the highest interfere to Aβ42 assembly. Moreover, effects of spatial conformation of COS monomers with different DPs on binding with Aβ42 should also be considered to explain the DP-dependent manner of COS intervention. Therefore, more studies are needed to reveal interaction (for example, binding sites and intermolecular forces, etc.) between COS and Aβ42, which would provide more insights into the underlying mechanism involved.

Many studies have shown that Aβ oligomers can induce neuronal loss and cognitive impairment via multiple pathways in the early stage of AD, including inducing neuron apoptosis, increasing oxidative stress and promoting neuroinflammation [38]. Recently, researchers have confirmed that COS possess good neuroprotective activities [19]. Wu et al. reported that COS and their derivatives may be able to inhibit apoptosis of neuronal cells in brain cells [39]. Hao et al. discovered that PC12 cells pretreated with per-acetylated chitosan oligosaccharides significantly inhibited glutamate-induced cell apoptosis through regulating elevation of the Bax/Bcl-2 ratio and activation of caspase-3 [40]. Moreover, water-soluble chitosan with high molecular weights could protect against cell apoptosis induced by serum starvation in human astrocytes [41]. In our study, the results showed that COS significantly alleviated SH-SY5Y cell apoptosis by Aβ42 induced cytotoxicity. Therefore, reduction of Aβ42 neurotoxicity by COS intervention via inhibiting aggregation of Aβ42 or dissociating toxic Aβ42 aggregates into nontoxic forms might be one of the mechanisms underlying their anti-apoptotic activities. In addition to cell apoptosis, activated microglia-mediated oxidative damage and neuroinflammation contribute to AD pathogenesis. Actually, Aβ oligomers accumulated in brains could dramatically increase the ROS levels and further lead to dysfunction of CNS [42]. According to our present data, the ROS levels induced by Aβ42 oligomers in microglial BV2 cells were increased significantly, which were counteracted by the presence of COS (Figure 8A,B). In AD, microglia are able to bind with soluble Aβ oligomers and Aβ fibrils via cell-surface receptors, including CD14, CD47, and Toll-like receptors [43,44], resulting in activation of microglia, further leading to produce numerous proinflammatory cytokines and chemokines [45]. Based on the previous investigation, increases of Aβ concentration in mice brains were associated with increased concentrations of proinflammatory cytokines, including TNFα, IL-6, and IL-1β [46]. Additionally, sustained exposure to Aβ and inflammatory cytokines could aggravate the pathological progression of AD. In parallel, our data showed that the transcriptional levels of pro-inflammatory cytokines, such as TNF-α, IL-1β, and IL-6, were significantly up-regulated by Aβ42. On the contrary, such inflammatory responses were effectively inhibited by COS treatment (Figure 8C). Therefore, the possible mechanism might be that COS modified Aβ42 suppressed the binding capacities of Aβ42 against cell-surface receptors of microglia, and then alleviated the activation of microglia and reduced the release of inflammatory cytokines significantly. Since microglial activation and neuroinflammation are complex processes, other possible mechanisms could not be excluded. For example, our further research demonstrated that COS administration could also regulate the phosphorylation levels of p38 in MAPK inflammatory signaling pathway in Aβ42 oligomers stimulated BV2 cells, thus inhibiting the secretion of inflammatory cytokines (data not show). The above observations suggested that COS can inhibit inflammation and oxidative stress induced by Aβ42 oligomers.

## 4. Materials and Methods

### 4.1. Materials

Lyophilized powder of Aβ42 (>95%) was purchased from Chinese Peptide Co. Ltd. (Hangzhou, China). Cyanine 7 NHS (*N*-hydroxy succinimide) ester was bought from Lumiprobe Corporation (Hunt Valley, MD, USA). Thioflavin T (ThT), 4′,6-diamidino-2-phenylindole (DAPI), dimethyl sulfoxide (DMSO), Congo red (CR), propidium iodide (PI), and 3-(4,5-dimethylthiazol-2-yl)-2,5-diphenyltetrazolium bromide (MTT) were obtained from Sigma-Aldrich (St. Louis, MO, USA). 1,1,1,3,3,3-Hexafluoro-2-propanol (HFIP) was purchased from Aladdin Biological Co. Ltd. (Shanghai, China). Hoechst 33342 was purchased from Thermo Fisher Scientific (Waltham, MA, USA). Dulbecco’s modified Eagle’s medium (DMEM), fetal bovine serum (FBS), and mixtures of penicillin and streptomycin for cell cultures and cytotoxicity studies were acquired from Gibco Invitrogen (Grand Island, NY, USA). All solutions were prepared with deionized water (18.2 MΩ cm^−1^) collected from a Milli-Q water purification system (Millipore, Burlington, VT, USA). Human neuroblastoma SH-SY5Y cells, mouse microglial BV2 cells, and mouse brain microvascular bEnd.3 cells were purchased from American Type Culture Collection Inc. (Manassas, VA, USA). All other chemicals with the highest purity were available from local sources.

### 4.2. Preparation of COS and COS Monomers with Different DPs

COS were prepared by enzymatic hydrolysis of chitosan as described previously with slight modification [47]. Briefly, chitosan was degraded by endo-chitosanase in lactic acid buffer (pH = 6.0) at 40 °C, which was screened by our lab and produced in *Pichia pastoris*. The percentages or concentrations of COS monomers with different DPs were determined by HPLC.

COS monomers with different DPs were purified using Interchim puriFLASH 4250 HPLC system (Interchim, Martinique, France) equipment with an Acchrom XAmide column (20 mm × 250 mm × 10 μm, Acchrom, Beijing, China). Mobile phase was composed of water (a) and acetonitrile (b) and 100 mM ammonium formate (c) with a gradient elution over 70 min: 5–50% (a), 85–40% (b) and 10% (c). The flow rate was 20 mL·min^−1^. The evaporation light-scattering detector was set to a probe temperature of Nev 55 °C and Eva 85 °C, and the nebulizer gas (nitrogen) was adjusted to 26 psi. The collected fractions were lyophilized by vacuum freeze-drying, and the purity was verified by HPLC-MS.

### 4.3. Trans-Well Model for Evaluating BBB Penetration of COS In Vitro

To establish the in vitro BBB trans-well model for evaluating BBB penetration of COS, a density of 5 × 10^5^ bEnd.3 cells was seeded onto the upper insert (PET membrane, 0.4 μm pore size, Falcon; Fisher Scientific) and allowed to grow for six days. The barrier function was evaluated by measuring the apparent permeability (*P_app_*) value of FITC-labeled dextran (10 kDa; 2 nM/mL) through the monolayer of brain microvascular bEnd.3 cells every day. *P_app_* was calculated using the equation below [48]:

*P_app_*(cm/s) = (1/*AC*_0_)/(*dQ*/*dt*) (1)

where *A* = area of mass transfer, *C*_0_ = donor concentration of reagent in the upper insert medium, and *dQ*/*dt* = transmembrane transportation rate.

The expression of occludin, a kind of tight junction protein, was also detected by immunofluorescence to evaluate the barrier function of BBB model in vitro. After monolayer cells grew for six days, the PET membrane of the insert was clipped off. After washed with PBS for three times, the membrane was fixed with 4% paraformaldehyde for 15 min, permeabilized with 0.5% Triton X-100 (Aladdin, Shanghai, China) for 10 min, and blocked with 5% bovine serum albumin (BSA) for 1 h at room temperature. The cells were labeled with occludin antibody (Alexa Fluor^®^ 594 conjugate, OC-3F10) (Thermo Fischer Scientific, Waltham, MA, USA) at 1 µg/mL in 1% BSA and incubated for 3 h at room temperature. Nuclei were stained with antifade mounting medium with DAPI. Laser scanning confocal microscopy (Olympus FV1000, Olympus Corporation, Tokyo, Japan) was applied for visualization.

To evaluate the BBB penetration of COS, SH-SY5Y cells were plated at a density of 1 × 10^5^ cells in a 24-well plate and the cells were cultured for another 12 h. FITC-labeled COS with or without GLUT1 inhibitor (WZB-117) were added to the upper insert. After incubated for 6 h, SH-SY5Y cells were washed twice with PBS, fixed with 4% paraformaldehyde and stained with antifade mounting medium with DAPI. Triplicates of independent experiments for each treatment were performed. Images were visualized using an inverted fluorescence microscope (Leica DMI4000 B, Leica Microsystems, Weztla, Germany). The fluorescent intensity was quantified by Image J software (Version 1.5.3, National Institutes of Health, Bethesda, MD, USA).

### 4.4. In Vivo Real-Time Imaging to Investigate the BBB Permeability of COS

Six-week old male BALB/c nude mice were purchased from Beijing HFK Bioscience Co., Ltd. (Beijing, China). All animal experiments were performed in accordance with the China Public Health Service Guide for the Care and Use of Laboratory Animals. Experiments involving mice and protocols were approved by Institutional Animal Care and Use Committee of Tsinghua University. COS were labeled on amino groups by Cy7 NHS ester according to the manufacturer’s instructions. Male BALB/c nude mice were administrated with Cy7-labeled COS by gavage (Cy7-labeled COS dose of 200 mg/kg) and Cy7 NHS ester was used as the control group. Distribution of Cy7-labeled COS in mice was studied by IVIS spectrum in vivo imaging system (PerkinElmer, Waltham, MA, USA). The fluorescent images were taken at 0.5 h, 2 h, 6 h, and 12 h post administration of Cy7-labeled COS or Cy7 NHS and mice were sacrificed at 12 h post administration. The brains were collected immediately after washed with saline, and visualized under an IVIS spectrum in vivo imaging system (PerkinElmer, Waltham, MA, USA).

### 4.5. Preparation of Aβ42 Monomers

Aβ42 was prepared as previously described with minor modification [49]. Briefly, lyophilized powder of Aβ42 was dissolved in HFIP to a final concentration 1.0 mg/mL. The solution was left still at least for 2 h at 4 °C and then sonicated for 20 min to remove any pre-existed Aβ aggregates. Thereafter, the solution was centrifuged at 14,000 rpm for 20 min at 4 °C. Finally, about 75% of the supernatant was collected and lyophilized by vacuum freeze-drying overnight. Before use, Aβ42 was stored at −80 °C temporarily and used within two weeks.

### 4.6. Inhibition and Sisruption Assay for Aβ42 Fibrils Intervened with COS

A homogeneous solution of Aβ42 monomers was required for inhibition and disruption tests. Purified Aβ42 powder was first dissolved in 20 mM NaOH by sonicating for 5 min. Thereafter, the solution was centrifuged at 14,000 rpm (4 °C) for 30 min and the supernatant was collected for inhibition and disruption experiments. The concentration of Aβ42 was determined by a BCA protein assay kit (Solarbio Science and Technology Co., Ltd., Beijing, China). Finally, Aβ42 was diluted to 20 μM in 10 mM Tris-HCl buffer (pH 7.4), followed by immediate vortexing to mix thoroughly.

For inhibition assay of Aβ42 fibrils, 20 mg/mL COS in 10 mM Tris-HCl buffer (pH 7.4) was diluted with freshly prepared 20 μM Aβ42 monomer solution to 200, 500, and 1000 μg/mL, respectively. The mixed Aβ42-COS samples were incubated for 72 h at 37 °C. As for the disruption assay, Aβ42 fibrils were prepared by incubating 20 μM Aβ42 monomers in 10 mM Tris-HCl buffer (pH 7.4) for 24 h, which is long enough to enable Aβ42 monomers to grow into mature fibrils. Then, 20 mg/mL COS in 10 mM Tris-HCl (pH 7.4) was diluted with solution of 20 μM Aβ42 fibrils to 200, 500 and 1000 μg/mL respectively. All samples for disruption assays were incubated at 37 °C for another 48 h.

Aβ42 fibrils were prepared by incubating 20 μM Aβ42 monomers in 10 mM Tris-HCl (pH 7.4) for 24 h, which is sufficiently long enough to enable Aβ peptides to grow into mature fibrils at a saturated state. For disruption assay of Aβ42 fibrils, 20 mg/mL COS stock solution in 10 mM Tris-HCl (pH 7.4) was diluted with solution of 20 μM Aβ42 fibrils to a final concentration of 200, 500 and 1000 μg/mL, respectively. All samples for disruption assays were incubated at 37 °C for another 24 h.

### 4.7. Thioflavine T (ThT) Fluorescence Assay and Spectral Shift Assay of Congo Red (CR)

Aβ42 fibrillization and disruption of Aβ42 fibrils in the presence or absence of COS were monitored by ThT fluorescence assay and spectral shift assay of CR, which were always used to determine the presence of amyloid aggregates. A 2 mM ThT stock solution was further diluted using Tris-HCl buffer (10 mM, pH 7.4) to reach a final concentration of 20 μM. For each assay, the fluorescence intensity was measured in triplicate by a fluorescence plate reader. The wavelengths of excitation and emission were 440 and 480 nm, respectively. To exclude influence of background fluorescence on the experimental results, the fluorescence intensity of the solution without Aβ42 was subtracted. All data were the averages of three independent readings for each sample.

Spectral shift assay of CR was also used to quantify Aβ aggregates as described previously [50] with minor modification. CR at the concentration of 10 μM (10 mM PBS buffer, pH 7.2) was added to the samples described above immediately before the assay. Based on the previous report, the ratio of CR to Aβ fibrils should not fall below 1:5 [50]. Therefore, 20 μM Aβ42, with or without different concentrations of COS, was used. CR and Aβ42 fibrils were incubated at room temperature for 15 min prior to spectral analysis and then the absorbance at 540 nm was measured. Three measurements were performed and the data were averaged.

### 4.8. Circular Dichroism (CD) Spectroscopy

Secondary structure changes of Aβ42 were detected by CD spectroscopy according to a previous study with slight modification [51]. In brief, Aβ42 monomers in 20 mM NaOH were diluted with 10 mM Tris-HCl buffer (pH 7.4) to a final concentration of 20 μM with or without different concentrations of COS (200, 500, and 1000 μg/mL). The concentrations used for COS monomers with different DPs in CD spectroscopy were consistent with theirs in the COS mixture determined by HPLC analysis. CD measurements were conducted on a J-810 spectropolarimeter (JASCO Inc., Tokyo, Japan) at room temperature in a 1 mm path length quartz cuvette. The spectra were collected within 190–260 nm at 0.1 nm intervals with a 1 nm bandwidth and a scan rate of 100 nm/min. The baseline (10 mM Tris-HCl buffer with and without COS) was subtracted from the result for each sample. Each spectrum is the average of three scans and the spectra were smoothed using the Jasco software FFT filter function and converted into molar ellipticity. The percentages of secondary structures of each sample were estimated using the Protein Secondary Structure Estimation Program (version 1.0, Jasco Corp., Tokyo, Japan)

### 4.9. MicroScale Thermophoresis (MST) Study

Aβ42 was labeled using the MonolithTM RED-NHS Protein Labeling Kit (NanoTemper Technologies, Munich, Germany) according to the manufacturer’s instructions. Labeled Aβ42 was used at a concentration of 4 μM, while non-fluorescent labeled COS as binding ligands were titrated in 1:1 dilution series (concentrations between 800 μM and 12.2 nM). The same procedures were repeated for COS monomers with different DPs, and the highest final concentrations for each COS monomer with different DPs were 8.7 mM DP3, 7.5 mM DP4, 0.6 mM DP5, and 0.5 mM DP6, respectively.

Samples were filled in the Monolith NT.115 MST standard-treated capillaries (NanoTemper Technologies, Munich, Germany) and immediately measured by MST using MO. The control software maintained the temperature at 37 °C after the samples were mixed well. All experimental parameters used by the MST instrument were adjusted to 20% LED power and 40% MST power. Triplicates of independently-pipetted measurements were analyzed. The MO-Affinity Analysis software (version 2.1.3, Nano Temper Technologies, Munich, Germany) was used to calculate the binding affinity expressed in term of the *K_D_* value.

### 4.10. Transmission Electron Microscopy (TEM)

Morphological changes of Aβ42 aggregates in the presence or absence of COS were characterized by TEM. Samples used in CD spectroscopy were diluted at a 1:4 ratio with 10 mM Tris-HCl buffer (pH 7.4). For each assay, 10 μL solution was adsorbed onto a glow-discharged, Formvar carbon-coated copper grid (200 mesh) for 5 min. The droplet was negatively stained with an equal volume of 1% glutaraldehyde (*v*/*v*) and incubated for an additional 5 min. The excess solution was blotted, and the grid was air-dried. The prepared samples were examined using a Hitachi H7500 TEM (Hitachi, Tokyo, Japan) at the voltage of 80 kV.

### 4.11. MTT Assay to Detect Cell Viability

Cells were plated in 96-well polystyrene plates with approximately 5000 cells/200 μL of medium per well. Plates were incubated at 37 °C for 24 h to allow cells to attach to plate surface. To determine cytotoxicity of COS, COS with different concentrations were co-incubated with human neuroblastoma SH-SY5Y cells, mouse microglial BV2 cells and mouse brain microvascular bEnd.3 cells for 12 h. Cells without COS intervention were set as the control group. As for detecting the protective effects of COS on Aβ42 oligomers induced cytotoxicity, Aβ42 oligomers (5 μM) mixed with or without different concentrations of COS were diluted with fresh medium and added to individual wells. The same volume of only medium was added to control cultures. The plates were then incubated for an additional 24 h at 37 °C.

Cell viability was determined by a mitochondria enzyme-dependent reaction of MTT. Briefly, MTT solution in fresh DMEM culture medium was added to a final concentration of 0.5 mg/mL. The plates were incubated at 37 °C for additional 4 h. Finally, the medium containing MTT was removed and 100 μL DMSO was added to each well, and the medium was agitated at room temperature for 30 min to dissolve crystals. The amount of formazan was determined by measuring the absorbance at 570 nm. Results were expressed as the percentages of the MTT reduction compared with the untreated group.

### 4.12. Detection of Cell Apoptosis

SH-SY5Y cells were plated in six-well polystyrene plate with approximately 10^6^ cells per well. Plate was incubated at 37 °C for 12 h to allow cells to attach to plate surface. A final concentration of 10 μM of Aβ42 was used to treat SH-SY5Y cells with or without COS at 200 μg/mL. For flow cytometry experiments, an Annexin V-FITC apoptosis detection kit (CA1040) (Solarbio Science and Technology Co., Ltd., Beijing, China) was used according to the manufacturer’s instructions. Briefly, cells were washed twice with pre-cooled PBS and trypsinized (EDTA-free), and centrifuged at 1000 rpm for 5 min at 4 °C. Cells were resuspended in binding buffer at a concentration of 1 × 10^6^/mL, where 100 μL was transferred to the test tube. Cells were incubated with 5 μL FITC- conjugated Annexin V and PI for 10 min at 37 °C in the dark. After addition of 400 μL binding buffer, samples were immediately analyzed by flow cytometry (CytoFLEX; Beckman, Brea, CA, USA).

For immunocytochemistry experiments, 5 × 10^4^ cells were seeded onto glass cover slips of 24-well plate with 0.5 mL of DMEM supplemented with 10% fetal bovine serum and 1% Penicillin-Streptomycin, and grown at 37 °C for 12 h. Cells were rinsed three times with PSB, and incubated with Hoechst 33342 at 5 μg/mL for 15 min in the dark. Meanwhile, PI was co-stained for necrotic cells. After then, cells were washed three times with PBS and detected on coverslips with anti-fade mounting medium (Solarbio Science and Technology Co., Ltd., Beijing, China). The percentage of apoptotic cells with condensed nuclei was quantified by Image J software (Version 1.5.3, National Institutes of Health, Bethesda, MD, USA).

### 4.13. DCFH-DA Detection of Cell Oxidative Stress

Oxidative stress state of cells was fluorometrically monitored using DCFH-DA. BV2 Cells, incubated with 10 μM Aβ42, were treated with or without 200 μg/mL COS at 37 °C for 12 h. Cells were washed three times with pre-cooled PBS. DCFH-DA was diluted in fresh DMEM (without phenol red) to a final concentration of 10 μM and then incubated with cells for 30 min in the dark at 37 °C. The medium was then removed, and cells were washed three times with PBS again. The fluorescence intensity was detected using an inverted fluorescence microscope (Leica DMI4000 B, Leica Microsystems, Weztla, Germany). ROS production was calculated by Image J software (Version 1.5.3, National Institutes of Health, Bethesda, MD, USA) as a relative percentage to the control group. All assays were performed in at least three individual experiments.

### 4.14. Quantitative Real-Time Reverse-Transcription PCR (qRT-PCR) Analysis

Total RNA was extracted from BV2 cell with TRIzon reagent according to the manufacturer’s instruction. RNA samples were detected for concentration and purity on a Nanodrop 2000 spectrophotometer (Thermo Fischer Scientific, Waltham, MA, USA). RNA (1 μg) was reverse transcribed to cDNA by using a HiFiScript cDNA Synthesis Kit (Beijing Cowin Biotech Co., Ltd., Beijing, China) according to the manufacturer’s protocol. The qRT-PCR was performed using an UltraSYBR Mixture Kit (Beijing Cowin Biotech Co., Ltd., Beijing, China) on an ABI StepOne^TM^ Real-time System (ABI, Carlsbad, CA, USA). The relative mRNA levels of TNF-α, IL-6 and IL-1β were normalized against that of β-Actin. The primer sequences were listed in Supplementary Table S2. The 2^−ΔΔCt^ method was used to calculate the relative quantification of transcription mentioned above.

### 4.15. Statistical Analysis

Data were presented as means ± SEM. The differences between two groups were compared using student’s t-test, one-way or two-way ANOVA followed by Tukey’s honestly significant difference post hoc test. Regular analysis was analyzed with GraphPad Prism 8.0.0 (GraphPad Software Inc., San Diego, CA, USA). In all cases, statistical significance was accepted at *p* < 0.05, and * indicates *p* < 0.05 and ** indicates *p* < 0.01, *** indicates *p* < 0.001, and **** indicates *p* < 0.0001.

## 5. Conclusions

In conclusion, our findings demonstrated that COS showed good BBB penetration ability in vivo and in vitro and GLUT1 was identified as one of the transporters accounting for their penetration of BBB. The underlying pharmacological mechanism of COS on alleviating Aβ42-mediated cytotoxicity might be associated with their direct binding ability with Aβ42. Owing to their corresponding properties, COS showed efficient inhibition effects on Aβ42 fibrillation and disaggregation effects on preformed Aβ42 fibrils in a dose- and DP-dependent manner. Meanwhile, COS significantly alleviated amyloid-induced apoptosis, oxidative stress and release of inflammatory cytokines (Figure 10). Our studies highlighted the potential role of COS as novel therapeutic agents for prevention or treatment of AD.

## Supplementary Materials

The following are available online at https://www.mdpi.com/1660-3397/18/10/488/s1, Table S1: Binding affinities and binding constants of COS monomers with different DPs and COS mixture against Aβ42. Table S2: Primers used in this study. Figure S1: ^1^H NMR spectrum of COS. Figure S2: Evaluation of permeability of the blood–brain barrier (BBB) in vitro. Figure S3: Cell viability detected by mitochondria enzyme-dependent reaction of MTT assay.
