# Supplementary figures and images for: Blood-Brain Barrier Permeable Chitosan Oligosaccharides Interfere with β-Amyloid Aggregation and Alleviate β-Amyloid Protein Mediated Neurotoxicity and Neuroinflammation in a Dose- and Degree of Polymerization-Dependent Manner
Source: Mar Drugs. 2020 Sep 25;18(10):488. doi: 10.3390/md18100488 (PMC7650801; doi:10.3390/md18100488)

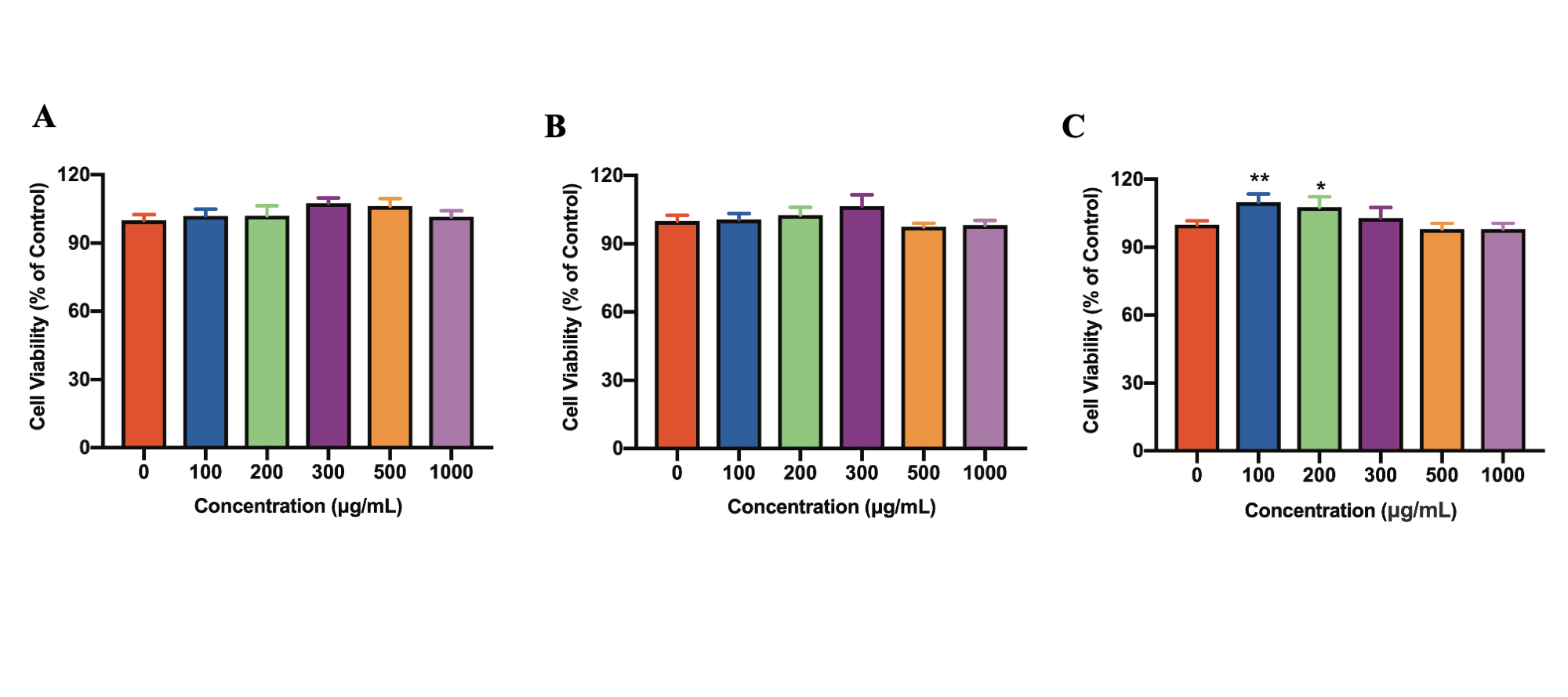

Supplement: Supplementary file 1 [file marinedrugs-18-00488-s001.zip › Revised supplementary materials/Figure S3.tiff]

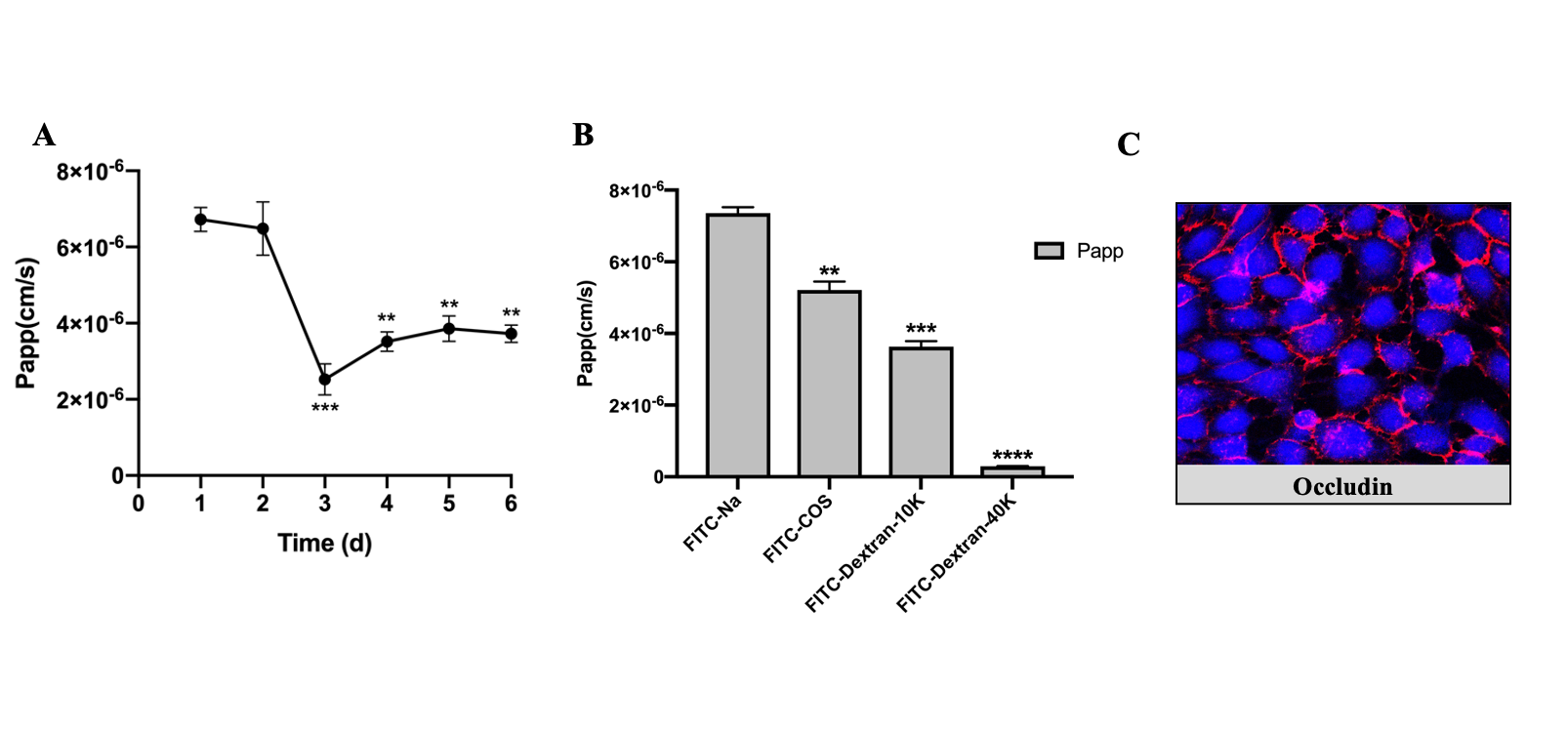

Supplement: Supplementary file 1 [file marinedrugs-18-00488-s001.zip › Revised supplementary materials/Figure S2.tiff]

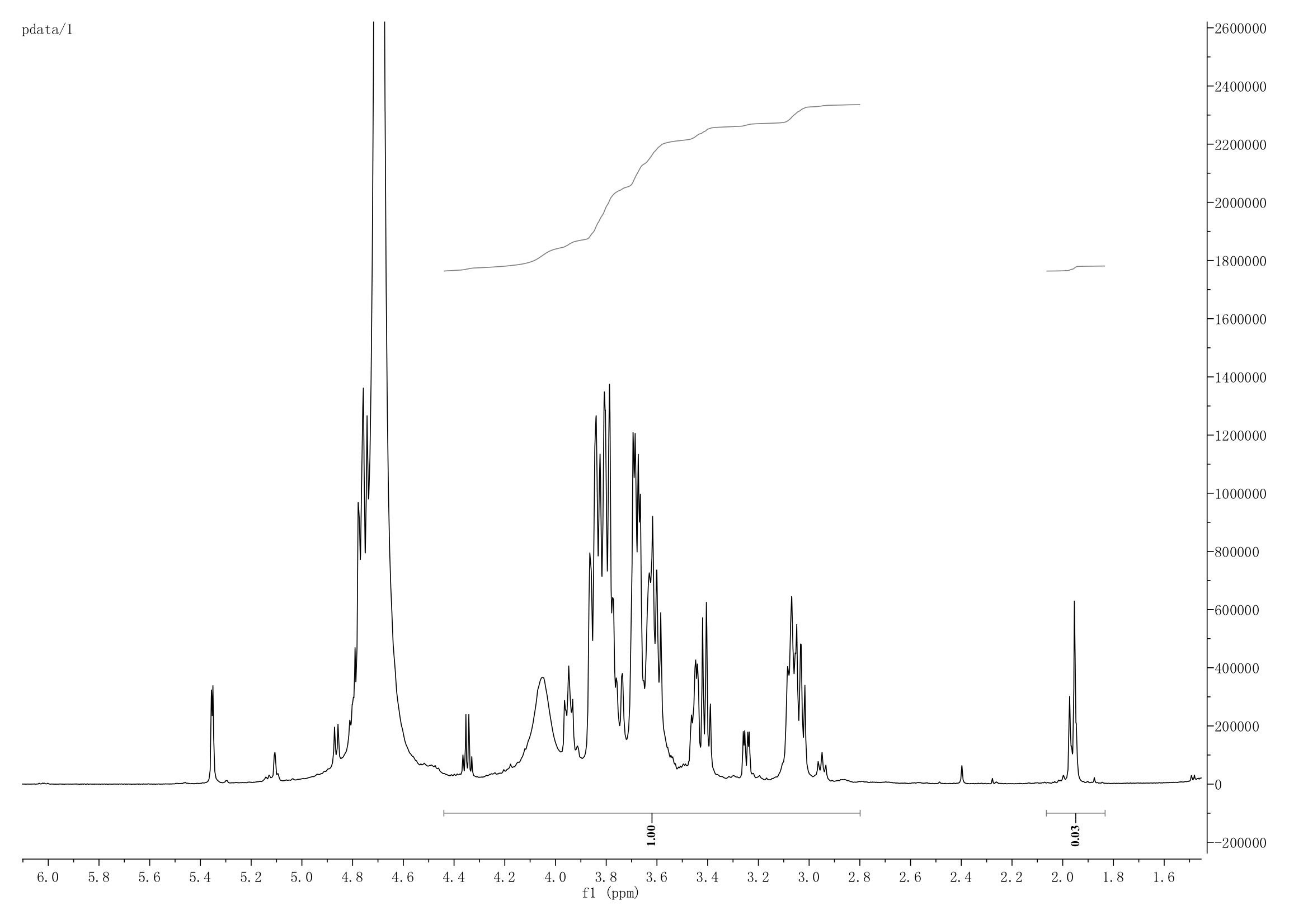

Supplement: Supplementary file 1 [file marinedrugs-18-00488-s001.zip › Revised supplementary materials/Figure S1.tif]
