# Supplementary material for: Blood-Brain Barrier Permeable Chitosan Oligosaccharides Interfere with β-Amyloid Aggregation and Alleviate β-Amyloid Protein Mediated Neurotoxicity and Neuroinflammation in a Dose- and Degree of Polymerization-Dependent Manner
Source: Mar Drugs. 2020 Sep 25;18(10):488. doi: 10.3390/md18100488 (PMC7650801; doi:10.3390/md18100488)
Supplement: Supplementary file 1 [file marinedrugs-18-00488-s001.zip › Revised supplementary materials/Revised Supplementary data.doc]

**Blood-brain-barrier permeable chitosan oligosaccharides** **interfere with** **β-amyloid aggregation and alleviate β-amyloid protein mediated neurotoxicity and neuroinflammation in a dose and** **degree of polymerization dependent manner**

Limeng Zhu^1,2,^ ^†^, Ruilian Li^1,2,^ ^†^, Siming Jiao^1^, Jinhua Wei^1^, Yalu Yan^1^, Zhuo A. Wang^1,*^, Jianjun Li^1,*^ and Yuguang Du^1,*^

^1^State Key Laboratory of Biochemical Engineering, Institute of Process Engineering, Chinese Academy of Sciences, Beijing 100190, China

^2^University of Chinese Academy of Sciences, Beijing 100049, China

^†^These authors contributed equally to this work.

*Corresponding Authors:

Institute of Process Engineering, Chinese Academy of Sciences, 1 North 2nd Street, Zhongguancun, Haidian District, Beijing, PR China

**Tel:** +86-10-8254-5070 (J.L. & Z.A.W. & Y.D.)

**E-mail:** jjli@ipe.ac.cn (J.L.); [wangzhuo@ipe.ac.cn](mailto:wangzhuo@ipe.ac.cn) (Z.A.W.) ygdu@ipe.ac.cn (Y.D.)

**Table of Contents**

1. Table S1. Binding affinities and binding constants of COS monomers with different DPs and COS mixture against Aβ42…………………………………………………………...…3
2. Table S2. Primers used in this study……………………………………………..….…..4
3. Fig. S1. ^1^H NMR spectrum of COS…………………………………………………......5
4. Fig. S2. Evaluation of permeability of the blood–brain barrier (BBB) in vitro…….…...6
5. Fig. S3. Cell viability detected by mitochondria enzyme-dependent reaction of MTT assay…………………………………………………………………………………..…7

**Supplementary tables**

**Table S1.**  Binding affinities and binding constants of COS monomers with different DPs and COS mixture against Aβ42

|  | **DP3** | **DP4** | **DP5** | **DP6** | **COS** |
| --- | --- | --- | --- | --- | --- |
| *K****_d_*** (μM) | 447 ± 196 | 168 ± 78 | 7.854 ± 1.03 | 2.09 ± 0.87 | 3.76 ± 0.34 |
| *K_a_* (M^-1^) | 2.24±0.98×10^3^ | 5.95±2.76×10^3^ | 1.3±0.17×10^5^ | 5.95±2.48×10^5^ | 2.66±0.24×10^5^ |

**Table S2.** Primers used in this study

| **Primers** | **Forward primer** | **Reverse primer** |
| --- | --- | --- |
| IL-1β | 5'-GCAACTGTTCCTGAACTCAACT-3' | 5'-ATCTTTTGGGGTCCGTCAACT-3' |
| IL-6 | 5'- TAGTCCTTCCTACCCCAATTTCC -3' | 5'-TTGGTCCTTAGCCACTCCTTC-3' |
| TNFα | 5'- CCCTCACACTCAGATCATCTTCT-3' | 5'- GCTACGACGTGGGCTACAG-3' |
| β-actin | 5'-AGGTGACAGCATTGCTTCTG-3' | 5'-GCTGCCTCAACACCTCAAC-3' |

**Supplementary figures**

**Fig. S1.** **^1^H** **NMR spectrum of COS.** The solution of COS for ^1^H NMR spectrum was prepared by dissolving 30 mg of COS in 1 mL of D_2_O. ^1^H NMR spectrum was acquired on an AVANCE III 600 MHz spectrometer (Bruker, Germany). The experiment was run at 70 °C at which the solvent (D_2_O) peak didn’t interfere with the signals of COS.

**Fig. S2.** **Evaluation of permeability of the blood–brain barrier (BBB) *in vitro*.** **(A)** Apparent permeabilities of bEnd.3 cells cultured for different periods in Transwell calculated from the diffusion of a paracellular marker (FITC-dextran; 10 k Da) with an endothelial monolayer. **(B)** Apparent permeabilities of different paracellular markers (Fluorescein sodium, FITC-COS, 10 k Da and 40 kDa FITC-dextran) through endothelial monolayer on day 6. **(C)** Immunofluorescence staining analysis of localization of tight junction protein occludin using Confocal laser microscope (Scale bars, 50μm). The continuous marginal localization of the tight junction protein occludin reflected the tightness of the BBB. Data were represented as mean ± SEM of three independent experiments. **p < 0.01, ***p < 0.001, ****p < 0.0001.

**Fig. S3. Cell viability detected by mitochondria enzyme-dependent reaction of MTT assay.** Cells were plated in 96-well polystyrene plates with approximately 5000 cells/200 μL of medium per well. Plates were incubated at 37 °C for 24 hr to allow cells to attach to the plate surface. COS with different concentrations were co-incubated with human neuroblastoma SH-SY5Y cells **(A)**, mouse microglial BV2 cells **(B)** and mouse brain microvascular bEnd.3 cells **(C)** for 12 hr. The amount of formazan was determined by measuring the absorbance at 570 nm. Results were expressed as the percentages of MTT reduction compared with untreated group. Error bars indicated SEM; n = 6; *p < 0.05, **p < 0.01.
